# Effect of acetaminophen on relieving orthodontic pain with clear aligner based on GAD-7: A retrospective research

**DOI:** 10.1016/j.heliyon.2023.e23292

**Published:** 2023-12-03

**Authors:** Yunan Gao, Rui Wang, Qiong Liu, Bo Zhou, Hu Qiao

**Affiliations:** a Key Laboratory of Shaanxi Province for Craniofacial Precision Medicine Research, College of Stomatology, Xi'an Jiaotong University, Xi'an, Shaanxi, China; b Respiratory and Critical Care Medicine, The First Affiliated Hospital of Xi'an Jiaotong University, Xi'an, Shaanxi, China

**Keywords:** Clear aligners, GAD-7, Acetaminophen, Orthodontic pain, Verbal behavior modification

## Abstract

**Objectives:**

Patients may have uncomfortable feelings during orthodontic treatment, which can directly lead to dissatisfaction. So in order to improve the patient's sense of pleasure during the treatment, it would be of great benefit if orthodontic pain can be relieved.

**Materials and methods:**

We included 150 patients wearing clear aligners from 18 to 30 years old during 2018–2020. Then designed following groups to determine the effectiveness of both verbal behavior modification and combination therapy with acetaminophen in reducing treatment pain: Group A, generalized anxiety disorder 7 (GAD-7) scored 0–4; Group B, GAD-7 scored 5–9; Group C, GAD-7 scored 10–14; and Group D, GAD-7 scored 15–21.

**Results:**

There was a difference in the visual analog scale (VAS) between verbal behavior modification with and without a 300-mg acetaminophen tablet oral QD in Group A (received the intervention at 8 h and 1 d), Group B at 8 h and 1 d, Group C at 8 h, 1 d, 2 d, and 3 d, and Group D at 8 h, 1 d, 2 d, 3 d, and 4 d. After 8 h, 1 d, 2 d, 3 d, and 4 d in patients with verbal behavior modification, VAS was markedly increased in Group D compared with Group A, B and C. Furthermore, after 8 h and 1 d in patients with verbal behavior modification and 300-mg acetaminophen tablet oral QD, VAS was strongly enhanced in Group D.

**Conclusions:**

Dental anxiety is strongly associated with pain in orthodontic patients receiving clear aligners. Acetaminophen administration may be a benefit in orthodontic pain that results from clear aligners, especially in the group with more GAD-7.

## Highlights

• Dental anxiety is close to orthodontic pain.
• Acetaminophen is effective on relieving pain from clear aligners.
• Only verbal behavior modification has limited effect for patients with more anxiety.

## 2 Introduction

In general, patients may discontinue medical treatment because of insufferable pain, which can directly affect the quality of life of patients [1,2]. In addition, as a subjective feeling, pain may be differentially expressed, depending on age, sex, cultural differences, emotional states, and various other factors [3,4]. Due to the fact that pain directly influences patient satisfaction during orthodontic treatment, methods of reducing pain can be greatly beneficial for patients [[5], [6], [7]].

Accordingly, orthodontic pain often occurs several hours after the exertion of force, and most orthodontic pain can be observed after 24 h; subsequently, at 7 days later, orthodontic pain can decrease to a near-baseline level [8]. Therefore, the first 7 days after treatment may serve a significant role during the entire treatment process. Some studies have demonstrated a regular pattern of pain in patients treated with fixed appliances and clear aligners [9,10].

Clear aligners are becoming more popular as an orthodontic treatment method than they have ever been [11,12]. There are several advantages associated with the use of clear aligners, such as comfort, aesthetics, and oral hygiene. Moreover, some results have shown that patients wearing clear aligners suffer less pain during the first 7 days of treatment than those patients wearing fixed appliances [13].

Dental anxiety is strongly associated with pain in orthodontic patients. Pain can be co-influenced by cognitive, environmental, and psychological factors, according to the gate-control theory. Anxiety, contained with psychological factors [14], can be strongly relevant to orthodontic pain [15]. Numerous medical and dental studies have identified the relationship between pain and anxiety [16]. Invisible orthodontic treatment is different from fixed appliances for the relatively instant and intermittent force. However, the specific effect of acetaminophen in alleviating the pain of patients with clear aligners during tooth movement is still not understood enough.

**Fig. 1:**
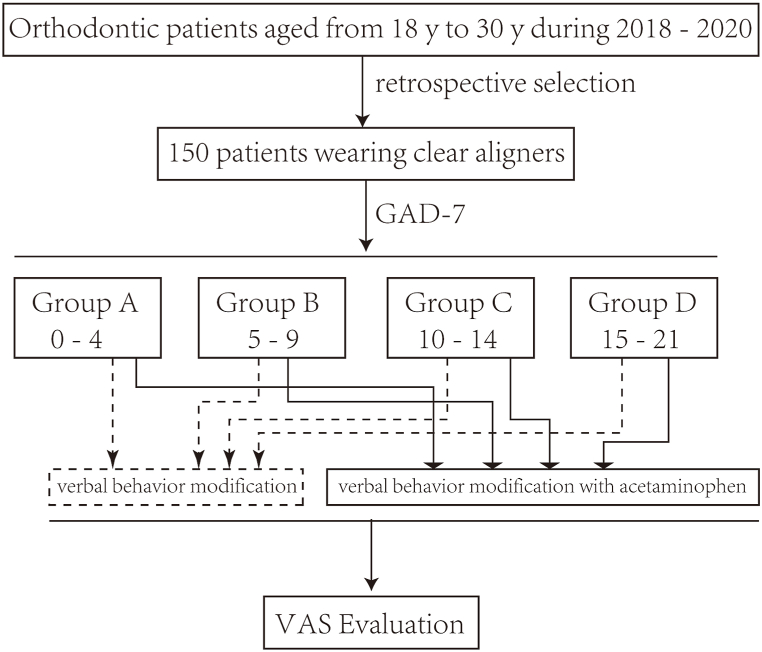
Flow chart of the retrospective study.

Based on the relevant studies [[17], [18], [19], [20]], we supposed that both verbal behavior modification and acetaminophen can relieve orthodontic pain effectively in patients with or without anxiety, the regular pattern and the specific effect of the relief, however, still remains unclear. Therefore in the present study, we evaluated the level of dental anxiety via generalized anxiety disorder 7 (GAD-7) scores and assessed the regular pattern and relevant level of pain by using visual analog scale (VAS) among groups that were divided according to GAD-7 scores who received clear aligners (Invisalign) with verbal behavior modification, either with or without 300-mg acetaminophen tid (Fig. 1.). The aim of this study was to determine whether there were any differences between verbal behavior modification and the combination with acetaminophen in reducing orthodontic pain among groups based on GAD-7 scores, which can provide clinical guidance to relieve pain and improve the patients' treatment comfort and confidence.

## 3 Materials and Methods

### 3.1 Subjects

This retrospective trial included a cohort of 150 patients wearing clear aligners (Invisalign) from Hospital of Stomatology Xi'a Jiaotong University between 2018 and 2020. Referred to other researches [17,21,22], we tried to get the sample size based on the inclusion criteria as much as possible, the base number was not large but we still obtained enlightenment. Written informed consent was obtained according to the Declaration of Helsinki, and was approved by the ethics committee of our university.

The following inclusion criteria were as follows.

(1) age between 18 and 30 years;
(2) dental space or crowdness problem;
(3) class Angle I/II/III misocclusion;
(4) absence of abnormal shape or size of tooth crown and root;
(5) access to complete follow-up.

In addition, the exclusion criteria were as follows.

(1) presence of pain from temporomandibular disorder (TMD) and/or other chronic craniofacial pain;
(2) pregnant/lactating women;
(3) patients with systemic disease;
(4) patients received orthodontic treatment before;
(5) poor compliance.

A GAD-7 (Generalized Anxiety Disorder-7) questionnaire was given to each patient immediately when they decided to start the treatment, and patients were required to complete the questionnaire on site. GAD-7 is one of the most common clinical measures for assessing anxiety disorders, included an inquiry points as follows: over the last 2 weeks, how often have you been bothered by the following problems? (1) feeling nervous, anxious or on edge, (2) not being able to stop or control worrying, (3) worrying too much about different things, (4) trouble relaxing, (5) being so restless that it is hard to sit still, (6) becoming easily annoyed or irritable, (7) feeling afraid as if something awful might happen. The response options were “not at all”, “several days”, “more than half the days”, and “nearly every day”, scored as 0, 1, 2, and 3, respectively. Higher score indicates higher levels of anxiety, specifically the level of anxiety severity GAD-7 scale score are as follows: minimal 0–4, mild 5–9, moderate 10–14, severe 15–21 [23]. Based on GAD-7 score, patients were divided into four groups: Group A: GAD-7 0–4 scores; Group B: GAD-7 5–9 scores; Group C: GAD-7 10–14 scores; Group D: GAD-7 15–21 scores.

After that, we compared verbal behavior modification with acetaminophen intervention and verbal behavior modification in different groups. The verbal behavior modification included a systematic inquiry points: (1) the patient's well-being, (2) whether pain and discomfort were present, (3) reassurance that the patient's reaction was within normal limits, (4) the necessity of sustained oral hygiene, (5) the need for a soft diet, (6) the use of analgesics, and (7) the importance of maintaining a positive attitude [24,25]. The verbal behavior modification was instructed at 8 h and 1 day after treatment. The patients with acetaminophen were administered 300-mg tid for 7 days according to the medicine instructions. The booklet with VAS (visual analog scale score) is given to the patients, and they need to fill it out at home after 8 h and 1, 2, 3, 4, 5, 6 and 7 days then return it to the doctors at the next appointment. A 10-cm VAS was designed to evaluate level of pain during treatment, and each millimeter represented a VAS score at 1, especially score 0 at the left end of VAS suggested no pain, meanwhile score 50 in the center suggested a moderate level, and score 100 at the right end suggested most severe pain level [26]. The VAS design has been fully explained to the patients before the research started.

### 3.2 Statistical analysis

Descriptive statistics were determined for the groups. Normality of the data was tested using Kolmogorov-Smirnov and Shapiro-Wilk tests. Data were not normally distributed; thus, the nonparametric Kruskal-Wallis, analysis of variance, and post hoc Mann-Whitney tests were used for analysis. As multiple analyses were conducted on the same dependent variable, there were greater chances of committing a Type I error, which was prevented using Bonferroni corrections. Spearman correlation was used to determine the correlation between the VAS score and GAD-7 score. All analyses were done using SPSS 17.0.

## 4 Results

### 4.1 Clinical characteristics of the enrolled subjects

In the process of orthodontic treatment, various age stages, such as adolescents, pre-adolescents, and adults may have different responses to pain, therefore we controlled the age stage to 18–30 years old, and considered gender may cause bias, we also tried to control gender differences between the groups, and finally enrolled 150 patients with invisible appliances referring to inclusion and exclusion criteria. We divided all 150 patients into the following groups: Group A, 52 subjects; Group B, 48 subjects; Group C, 29 subjects; and Group D, 21 subjects. The baseline information of the enrolled participants is shown in Table 1. No significant differences in age or sex were observed among the groups. Towards the anxiety level, we found that patients with GAD-7 15–21 showed higher anxiety tendency in clinical work, such as being more prone to pain, and worrying more about the treatment, even affecting sleep, and so on. In addition, in each group, we planned to compare verbal behavior modification with acetaminophen intervention and verbal behavior modification.

**Table 1:** Baseline information of the patients enrolled.

	Group A	Group B	Group C	Group D
N	52	48	29	21
Age y	22.3	26.7	22.5	25.4
Sex, No				
Female	37	37	19	17
Male	15	11	10	4
Ethnicity	Asian	Asian	Asian	Asian

#### 4.1.1 The influence of acetaminophen intervention on orthodontic pain

**Fig. 2:**
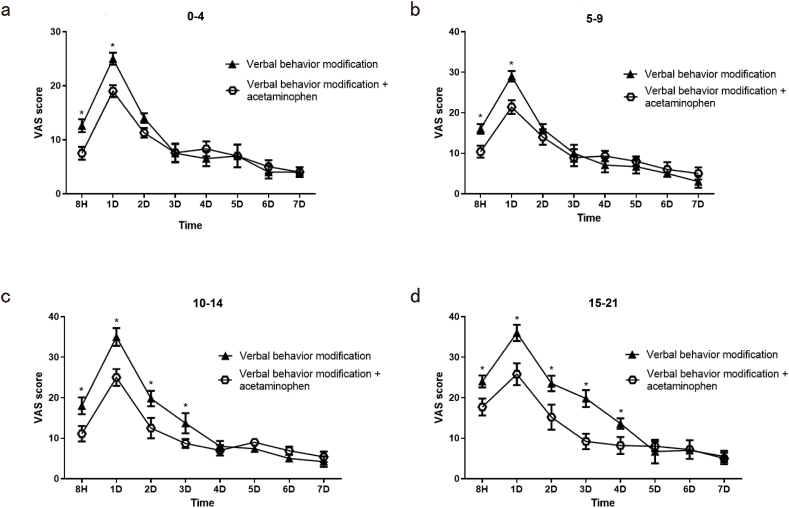
The influence of acetaminophen intervention on orthodontic pain. a. VAS scores in Group A (GAD-7 0–4 scores) with both verbal behavior modification and 300-mg acetaminophen tid compared with verbal behavior modification; b. VAS scores in Group B (GAD-7 5–9 scores) with both verbal behavior modification and 300-mg acetaminophen tid compared with verbal behavior modification; c. VAS scores in Group C (GAD-7 10–14 scores) with both verbal behavior modification and 300-mg acetaminophen tid compared with verbal behavior modification; d. VAS scores in Group D (GAD-7 15–21 scores) with both verbal behavior modification and 300-mg acetaminophen by mouth tid compared with verbal behavior modification. (*P < 0.05).

After daily acetaminophen intervention and verbal behavior modification in Group A, VAS scores were statistically decreased in the patients with acetaminophen intervention and verbal behavior modification, when compared to the use of only verbal behavior modification, after 8 h and 1 d (Fig. 2a.). Similarly, there was a statistically significant difference in VAS scores between verbal behavior modification with/without 300-mg acetaminophen tid in Group B receiving interventions at 8 h and 1 d, Group C at 8 h, 1 d, 2 d, and 3 d, and Group D at 8 h, 1 d, 2 d, 3 d, and 4 d (*P* < 0.05) (Fig. 2b–d.). Thus, acetaminophen can relieve orthodontic pain, especially in patients with higher GAD-7 scores.

##### 4.1.1.1 The influence of dental anxiety on orthodontic pain

**Fig. 3:**
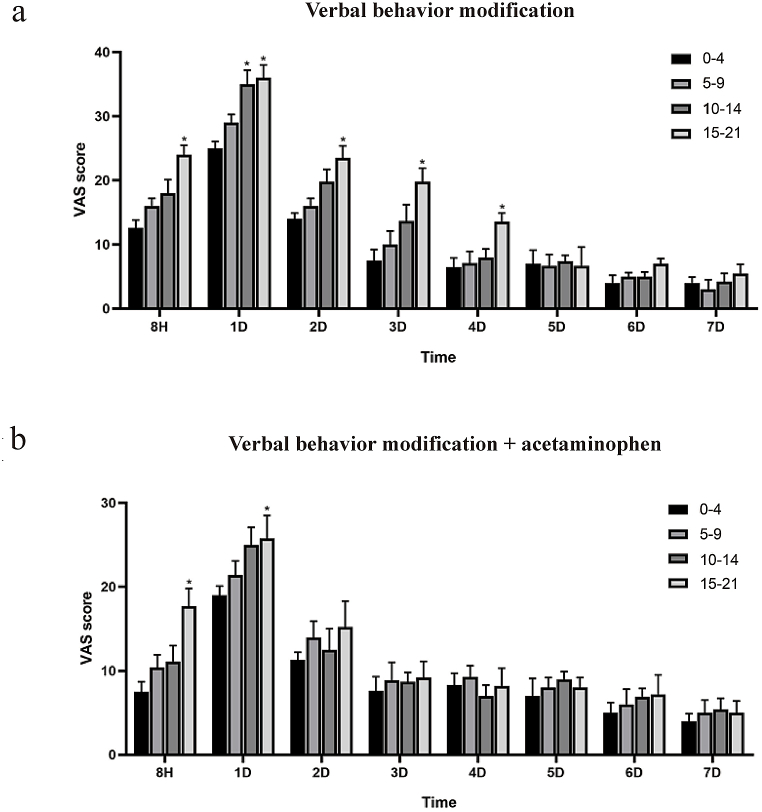
The influence of dental anxiety on orthodontic pain. a. After 8 h, 1 d, 2 d, 3 d, 4 d, 5 d, 6 d and 7 d in the patients receiving clear aligners with verbal behavior modification, VAS scores in Group A, Group B, Group C and Group D; b. After 8 h, 1 d, 2 d, 3 d, 4 d, 5 d, 6 d and 7 d in the patients receiving clear aligners with both verbal behavior modification and acetaminophen, VAS scores in Group A, Group B, Group C and Group D. (*P < 0.05).

**Fig. 4:**
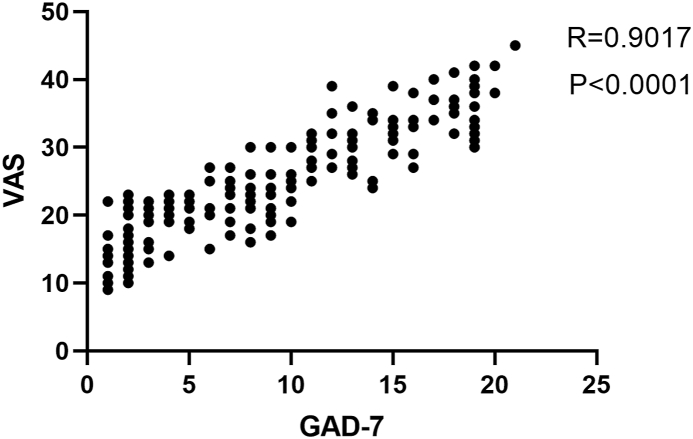
Correlation between the VAS score and GAD-7 score. Spearman correlation revealed significant correlation between the VAS score and GAD-7 score (R = 0.9017, P < 0.0001) groups.

After 8 h, 1 d, 2 d, 3 d, and 4 d in the patients receiving clear aligners with verbal behavior modification, VAS scores were markedly increased in Group D (Fig. 3a). Furthermore, a considerable difference was found in VAS scores among groups with both verbal behavior modification and 300-mg acetaminophen tid after 8 h and 1 d (*P* < 0.05) (Fig. 3b). There is no statistical difference in VAS scores between verbal behavior modification with/without 300-mg acetaminophen tid, after the 4 day. Thus, GAD-7 is strongly associated with VAS, which indicates that there is a direct influence of dental anxiety on orthodontic pain. Additionally, acetaminophen can exhibit therapeutic benefits in orthodontic pain that results from the use of clear aligners, especially in the group with more GAD-7. Since the pain peaked at 1 day, we show the details on 1 d in Table 2. A positive and significant correlation between VAS score and GAD-7 score was observed (R = 0.9017, *P* < 0.0001) groups were shown in Fig. 4.

**Table 2:** Mean pain scores (mm) at 1 d during clear aligners treatment.

	Verbal behavior modification				Verbal behavior modification with acetaminophen			
	Group A	Group B	Group C	Group D	Group A	Group B	Group C	Group D
Score	25.0	29.0	35.0	36.0	19.0	21.4	25.0	25.8
SD	1.1	1.3	2.2	2.0	1.1	1.7	2.1	2.7
95 % CI	23.9–26.1	27.7–30.3	32.8–37.2	34.0–38.0	17.9–20.1	19.7–23.1	22.9–27.1	23.1–28.5

## 5 Discussion

In this retrospective trial, we first evaluated the effectiveness of acetaminophen on orthodontic pain control in patients with more or less anxiety wearing invisible appliances. There was a statistically significant difference in VAS scores between verbal behavior modification with/without 300-mg acetaminophen tid in Group A receiving intervention at 8 h and 1 d, Group B at 8 h and 1 d, Group C at 8 h, 1 d, 2 d, and 3 d, and Group D at 8 h, 1 d, 2 d, 3 d, and 4 d. Thus, verbal behavior modification with acetaminophen intervention was shown to be more effective than the use of verbal behavior modification alone in all of the subjects. Moreover, the patients receiving clear aligners with acetaminophen exhibited more therapeutic benefits in reducing orthodontic pain, especially in the group with higher GAD-7 scores. Similar to our results, several randomized, single-blind, placebo-controlled trials have shown that both the psychological intervention and acetaminophen intervention groups had significantly less pain than only the psychological intervention group on orthodontic pain. Therefore, acetaminophen is an effective drug for reducing orthodontic pain that is due to the use of clear aligners, especially in patients with higher GAD-7 scores.

Second, our study showed that, after 8 h, 1 d, 2 d, 3 d, and 4 d in the patients receiving clear aligners with verbal behavior modification, VAS scores were markedly increased in Group D, when compared with Group A, Group B, and Group C. In addition, after 8 h and 1 d in the patients receiving clear aligners with verbal behavior modification and a 300-mg acetaminophen tid, VAS scores were strongly enhanced in Group D. Thus, higher GAD-7 scores indicated higher VAS scores in the groups with not only verbal behavior modification, but also both verbal behavior modification and acetaminophen, thus indicating that GAD-7 is strongly associated with VAS and that there is a direct influence of dental anxiety on orthodontic pain. Moreover, acetaminophen can exhibit therapeutic benefits in orthodontic pain that is due to the use of clear aligners, especially in the group with higher GAD-7 scores. Consistent with our findings, several previous results have also shown that dental anxiety has an effect on orthodontic pain. Thus, especially in the group with GAD-7 15–21 scores, we should provide both psychological interventions and acetaminophen interventions.

Some limitations must be considered in this study. There are many factors affecting pain, such as age, gender, and the severity of malocclusion during the orthodontic process. In this study, the age and gender factors were balanced as much as possible. But different age may have different reaction to pain, so our research could not gain useful information about other age groups. Meanwhile, Our research referred to reports which showed that the degree of malocclusion does not have a significant impact on the generation of orthodontic pain [21,27], so the impact of similar level of severity of malocclusion on orthodontic pain was not mentioned. In our study, patients replaced the invisible appliances personally. But it's a pity that our research did not record the replacement frequency for each group of patients. Above on the current condition of existing patient data, this research failed to distinguish the difficulty of wearing invisible aligners. Thus follow-up research can continue to include the number of cases to expand the sample size, so as to better clarify that the severity of malocclusion is one of the factors of orthodontic pain or just a confounding factor in patients wearing clear aligners, and the conclusions may be more trustworthy with a larger sample size. Meanwhile, this study was a retrospective trial and could not completely reflect the effectiveness of acetaminophen intervention. A randomized, single-blind, placebo-controlled trial should be designed in the future. Finally, the methods of psychological intervention and acetaminophen intervention require further detailed studies.

## 6 Conclusion

The present results have identified the effectiveness of acetaminophen on orthodontic pain in patients receiving clear aligners, regardless of GAD-7 scores. As was obvious from our results, the patients receiving clear aligners with acetaminophen exhibited more therapeutic benefits in reducing orthodontic pain in the group with higher GAD-7 scores. Furthermore, we found that GAD-7 is strongly associated with VAS, as well as the fact that there is a direct influence of dental anxiety on orthodontic pain, thus suggesting that acetaminophen can exhibit therapeutic benefits in orthodontic pain that is due to the use of clear aligners, especially in the group with GAD-7 15–21 scores.

## Funding

This study was supported by 10.13039/501100001809National Natural Science Foundation of China (Program No. 82001087), Key 10.13039/100006190Research and Development Program of Shaanxi (Program No. 2020 KW-049) and the Fundamental Research Funds for the Central Universities (xzy012019104).

## Data availability

Data will be made available on request.

## CRediT authorship contribution statement

**Yunan Gao:** Writing – review & editing, Writing – original draft, Methodology, Investigation, Data curation, Conceptualization. **Rui Wang:** Writing – review & editing, Investigation, Data curation. **Qiong Liu:** Writing – review & editing, Writing – original draft, Investigation. **Bo Zhou:** Writing – review & editing, Writing – original draft, Supervision, Methodology, Conceptualization. **Hu Qiao:** Writing – review & editing, Writing – original draft, Visualization, Validation, Supervision, Resources, Project administration, Methodology, Investigation, Funding acquisition, Formal analysis, Data curation, Conceptualization.

## Declaration of competing interest

The authors declare that they have no conflicts of interest.
